# Genome-Wide Identification, Expression and Interaction Analyses of *PP2C* Family Genes in *Chenopodium quinoa*

**DOI:** 10.3390/genes15010041

**Published:** 2023-12-27

**Authors:** Dongdong Yang, Xia Zhang, Meng Cao, Lu Yin, Aihong Gao, Kexin An, Songmei Gao, Shanli Guo, Haibo Yin

**Affiliations:** 1 College of Life Sciences, Yantai University, Yantai 264005, China; 19353512768@s.ytu.edu.cn (D.Y.); zhangxia@ytu.edu.cn (X.Z.); caomeng123@s.ytu.edu.cn (M.C.); yinlu0127@s.ytu.edu.cn (L.Y.); gahgah123@s.ytu.edu.cn (A.G.); aankexin@s.ytu.edu.cn (K.A.); gsm021223@163.com (S.G.); 2 College of Grassland Sciences, Qingdao Agricultural University, Qingdao 266109, China; 3 High-Efficiency Agricultural Technology Industry Research Institute of Saline and Alkaline Land of Dongying, Qingdao Agricultural University, Dongying 257300, China; 4 Key Laboratory of National Forestry and Grassland Administration on Grassland Resources and Ecology in the Yellow River Delta, Qingdao Agricultural University, Qingdao 266109, China

**Keywords:** Protein phosphatase 2C (PP2C), *Chenopodium quinoa*, genome-wide, abiotic stress

## Abstract

Plant protein phosphatase 2Cs (PP2Cs) function as inhibitors in protein kinase cascades involved in various processes and are crucial participants in both plant development and signaling pathways activated by abiotic stress. In this study, a genome-wide study was conducted on the *CqPP2C* gene family. A total of putative 117 *CqPP2C* genes were identified. Comprehensive analyses of physicochemical properties, chromosome localization and subcellular localization were conducted. According to phylogenetic analysis, *CqPP2Cs* were divided into 13 subfamilies. *CqPP2Cs* in the same subfamily had similar gene structures, and conserved motifs and all the CqPP2C proteins had the type 2C phosphatase domains. The expansion of *CqPP2Cs* through gene duplication was primarily driven by segmental duplication, and all duplicated *CqPP2Cs* underwent evolutionary changes guided by purifying selection. The expression of *CqPP2Cs* in various tissues under different abiotic stresses was analyzed using RNA-seq data. The findings indicated that *CqPP2C* genes played a role in regulating both the developmental processes and stress responses of quinoa. Real-time quantitative reverse transcription PCR (qRT-PCR) analysis of six *CqPP2C* genes in subfamily A revealed that they were up-regulated or down-regulated under salt and drought treatments. Furthermore, the results of yeast two-hybrid assays revealed that subfamily A CqPP2Cs interacted not only with subclass III CqSnRK2s but also with subclass II CqSnRK2s. Subfamily A CqPP2Cs could interact with CqSnRK2s in different combinations and intensities in a variety of biological processes and biological threats. Overall, our results will be useful for understanding the functions of *CqPP2C* in regulating ABA signals and responding to abiotic stress.

## 1. Introduction

Protein phosphorylation and dephosphorylation are the main forms of reversible post-translational modifications, which control the important regulatory mechanisms of many biological processes by regulating the localization, conformation, stability, and activity of substrate proteins in eukaryotes [1]. The phosphorylation state of proteins is dynamically controlled by protein kinase (PK) and protein phosphatase (PP), where protein kinase transfers the phosphate group of donor ATP to the side chain of receptor protein, while protein phosphatase dephosphorylates phosphoprotein. According to their mechanism of catalysis, substrate specificity and specific response to inhibitors, eukaryotic PPs can be divided into protein tyrosine (Tyr) phosphatases (PTP), phosphoprotein phosphatase (PPP), metallo-dependent protein phosphatase (PPM), and aspartate (Asp)-dependent phosphatase [2]. The PTP family includes Tyr specific phosphatases (PTPs) and dual-specificity phosphatase (DsPTP) that dephosphorylates serine (Ser), Thr (threonine), and Tyr phosphoresidue. The PPP family consists of seven members: PP1, PP2A, PP2B, and PP4/5/6/7 [2]. The PPM family includes protein phosphatase 2C (PP2C), pyruvate dehydrogenase phosphatase, and other magnesium (Mg^2+^)/manganese (Mn^2+^)-dependent STPs [3].

PP2Cs widely exist in prokaryotes and eukaryotes, are evolutionarily conserved, and significantly regulate stress signal pathways [4]. The relatively conserved catalytic domain in eukaryotic PP2C protein is located at the N- or C-terminus, while the region of the non-catalytic domain is not highly conserved and has diverse amino acid sequences with different functions. The non-catalytic domain region is important for defining the function of PP2C members, as it contains sequence motifs and/or transmembrane regions related to cellular signaling, including those that interact with protein kinases [5,6]. PP2Cs, as negative regulators of protein kinase cascades activated in different processes, participate in regulating signaling pathways. In fission yeast, genetic evidence has shown that PP2Cs are involved in the negative regulation of osmotic sensing signals transmitted through the Wis1-MAPK cascade [7]. In budding yeast, two PP2Cs, PTC1 and PTC3, are negative regulators of the PBS2-HOG1 MAPK pathway. In the yeast HOG pathway, four types of PP2C phosphatases Ptc1-Ptc4 dephosphorylated differently two activated phosphorylation sites of Pbs2 MAP2K [8]. In humans, PP2Calpha negatively regulates the stress-responsive MAPK cascades through dephosphorylation and inactivation of MKK6, SEK1, and MAPK (p38) [9], while PP2Cbeta dephosphorylates and inactivates the MAPKK kinase TAK1 to negatively regulate the TAK1 stress-signaling pathway [10] and PP2Cepsilon associates stably with TAK1 and dephosphorylates TAK1 to inhibit the TAK1 signaling pathway [11]. *PpABI1A* and *PpABI2B* of group A *PP2C* are directly involved in ABA response, acting downstream of ABA-activated kinase and regulating ABA-induced genes in the moss *Physcomitrella patens* [12].

To date, extensive research has been conducted on *PP2C* family genes across various plant species. In the investigation of *Arabidopsis*, six members (ABI1, ABI2, AHG1, HAB1, HAB2 and AHG3) belonging to the A subfamily have been confirmed as co-receptors for abscisic acid (ABA). These genes play a negative regulatory role in the ABA signaling pathway [13]. The MAPK phosphatase AP2C1 of the PP2C subfamily B interacts with MPK3, MPK4, and MPK6 to control their activity [14]. The expression of *AP2C1* and the accumulation of AP2C1 protein are strongly and locally enhanced at the induction site of the syncytium, indicating that AP2C1 acts as a negative regulatory factor for MAPK (MPK3, MPK4, and MPK6) to ensure inhibition of MAPK activation in the developing syncytium [15,16]. POL-LIKE1 and POLTERGEIST encode the related protein phosphatases 2C of the PP2C subfamily C, which are crucial for the establishment of shoot and root meristem tissues during embryogenesis and the maintenance of stem cell pools during post-embryonic development in *Arabidopsis* [17,18]. *AtPP2C* of subfamily D participates in the response to saline and alkali stresses [19]. Within subfamily E, AtPP2C-6-6 engages in interactions with the histone acetyl transferase AtGCN5, contributing to the regulation of transpiration through the modulation of stomatal signaling [20]. In subfamily F, WIN2 plays a role in modulating plant defense by interacting with the bacterial effector HopW1-1 [21]. Likewise, the protein phosphatase homologue 1 (PPH1) of unclustered PP2Cs involves maintaining efficient photosynthesis through dephosphorylation of Lhcb1 and Lhcb2 in plants [22]. In rice, OsPP2C09 (Os01g62760) of subfamily A PP2C interacts with RING-H2 type E3 ligase OsRF1 to participate in the salt tolerance of rice [23]. SAL1 encodes PP2C. D phosphatase is located on the plasma membrane and can interact with PM H^+^- ATPase to inhibit its activity, participating in rice aluminum resistance [24]. The subfamily F PP2C phosphatase ZmPP84 participates in regulating drought stress responses by dephosphorylating ZmMEK1 to inhibit its kinase activity in maize [25]. Likewise, the subfamily B PP2C phosphatase ZmPP2C26 can dephosphorylate ZmMAPK3 and ZmMAPK7, participating in the negative regulation of drought tolerance in maize [26]. All these researches indicate that PP2Cs have multiple functions and are worthy of further research.

Quinoa (*Chenopodium quinoa* wild.) is one of the most nutritious cultivated crops in the world. At the same time, it exhibits strong resistance to various soil and climatic conditions, which allows quinoa to be planted on marginal land. *PP2C* is a multifunctional gene that regulates plant growth, development, and stress response and has been analyzed in many plants [27]. Despite this, the *PP2C* gene family in quinoa has not been explored. This study conducted a comprehensive genome-wide analysis of the *CqPP2C* gene family in quinoa, including gene identification, chromosomal localization, phylogenetic relationships, gene structures, conserved motifs and domains, gene duplication analysis, cis-acting elements analysis, and relative expression of *CqPP2C* genes. Furthermore, we conducted an analysis to explore potential interactions between subfamily A CqPP2Cs and CqSnRK2s, both of which respond to abscisic acid and abiotic stress. These results will provide important information for understanding the mechanisms of PP2C in abiotic stress signal transduction.

## 2. Results

### 2.1. Identification and Basic Information of PP2C Genes in Quinoa

A total of 121 PP2C-coding candidate genes were identified via BLASTP and HMM searching in quinoa (*Chenopodium quinoa*). By using the CDD program with default settings, it was found that 4 of the 121 candidate PP2Cs did not contain PP2C catalytic domains. Therefore, 117 genes in quinoa were identified as members of the PP2C family and labeled as *CqPP2C1* to *CqPP2C117* based on their order on chromosomes. The gene name, gene ID, chromosome location, number of amino acids (aa), molecular weight (Mw), isoelectric point (pI), instability index, hydrophilic coefficient, and subcellular localization prediction of 117 PP2C proteins were analyzed (Table S1). The lengths of proteins ranged from 110 aa residues (CqPP2C94) to 1501 aa residues (CqPP2C101). The Mw ranged from 11,495.04 kDa (CqPP2C94) to 167,616.03 kDa (CqPP2C101) and pI varied from 4.08 (CqPP2C8) to 9.44 (CqPP2C70). According to the instability index, it was determined that 62.3% of CqPP2Cs exhibit protein instability. Except for CqPP2C7, CqPP2C94 and CqPP2C108, all other CqPP2C showed GRAVY below zero, indicating that these proteins are hydrophilic. The results of subcellular localization prediction showed that most of the quinoa PP2Cs might be located in the cytoplasm, chloroplast, or nucleus. In addition, only CqPP2C6 might be located in the plasma membrane, CqPP2C7 and CqPP2C83 might be located in the mitochondria, CqPP2C51, CqPP2C72, CqPP2C73 and CqPP2C99 might be located in the endoplasmic reticulum, and CqPP2C57 might be located in the vacuole membrane. These results indicated that CqPP2C proteins were randomly distributed in cells and played a role in various environments.

### 2.2. Phylogenetic Analysis of CqPP2C Genes

**Figure 1:**
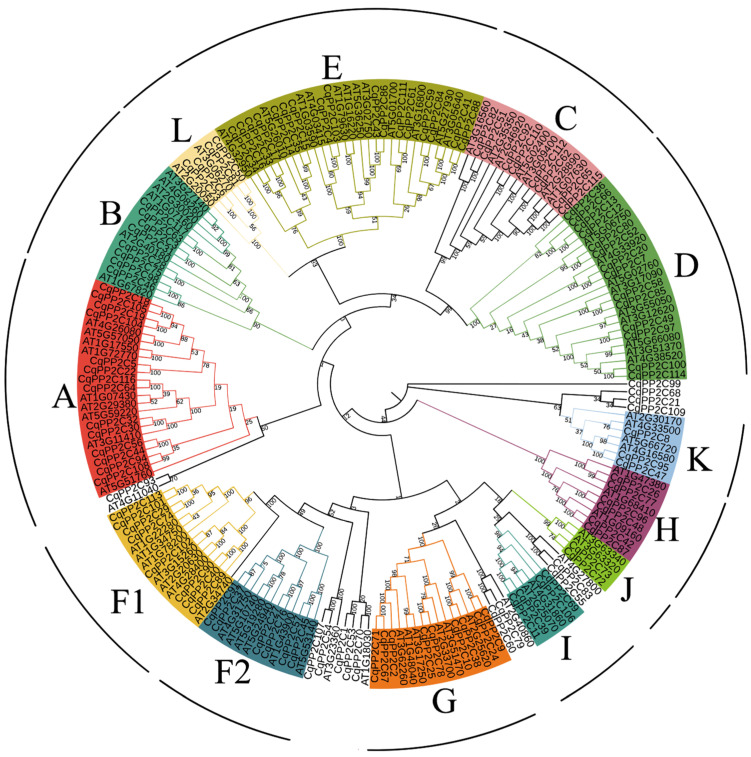
Phylogenetic analysis of PP2C proteins among *C. quinoa* and *Arabidopsis*.

To study the phylogenetic relationships between *PP2C* genes in quinoa and *Arabidopsis*, we used the maximum likelihood method to construct a phylogenetic tree based on the alignments of 80 PP2C protein sequences in *Arabidopsis* and 117 in quinoa (Figure 1).

The results showed that 103 CqPP2C proteins were divided into 13 subfamilies (A-L), including A (14), B (8), C(8), D (14), E (17), F1(8), F2 (6), G (9), H(6), I (4), J (2), K(3), L (4) (Table S2). In addition, quinoa has seven separate branches. The phylogenetic analyses indicated that *CqPP2C1*, *CqPP2C53*, *CqPP2C70* and *AT1G18030* tend to form independent branches. *CqPP2C60*, *CqPP2C79* and *AT2G40860* tend to form an independent branch; *CqPP2C55*, *CqPP2C83* and *AT4G27800* tend to form independent branches; *CqPP2C107*, *CqPP2C54* and *AT3G23360* tend to form independent branches; *CqPP2C93* and *AT4G11040* tend to form independent branches; *CqPP2C21*, *CqPP2C68*, and *CqPP2C109* tend to form independent branches; *CqPP2C99* tends to form independent branches.

### 2.3. Gene Structural and Conserved Domain Analyses of CqPP2Cs

**Figure 2:**
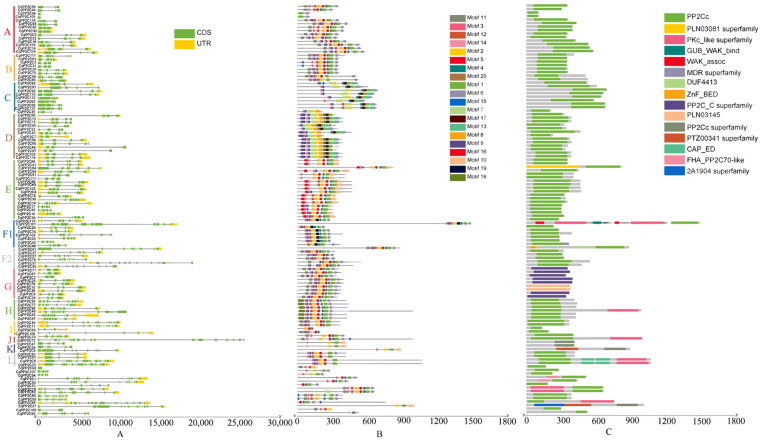
Gene structure, conserved motifs and domains of quinoa CqPP2Cs. (**A**) Gene structures of *CqPP2C* genes, yellow regions, green regions and black lines represent UTR, CDS and introns, respectively. (**B**) Conserved motif distribution of CqPP2C proteins; different color modules represent different motifs. (**C**) conserved domains of CqPP2C proteins.

We analyzed the exon/intron structure patterns of *CqPP2C* genes and protein-conserved motifs. In quinoa, *CqPP2Cs* contain a range of exons from 1 to 18. Thereinto, *CqPP2C93* in unclustered *PP2Cs* and *CqPP2C94* and *CqPP2C108* in subfamily A only had one exon without intron, while *CqPP2C72* in subfamily J had 18 exons and 17 introns, and had the longest intron. Generally, most genes in the same subfamily share a similar exon/intron structure (Figure 2A).

By employing the MEME motif search tool, we identified twenty motifs in the CqPP2C proteins. As illustrated in Figure 2B, the number of motifs varied between 3 and 11, encompassing 8 to 50 residues across all CqPP2C proteins. Among them, 107 CqPP2C proteins all contained motif 2. Each group had specific motifs, except for common motifs. For example, motifs 8 and 9 existed in group D but not in other groups, motifs 13 and 17 only existed in groups C and D, motifs 18 only existed in group H, and motifs 19 only existed in group F1. The distribution pattern of protein motifs from a group of family members was similar, with the G family having exactly the same motif distribution, indicating that CqPP2C members in the same cluster might have similar functions.

We used the NCBI CDD/SPARCLE database to predict conservative structural domains (Figure 2C). Most CqPP2Cs contain the PP2Cc domain, with only nine members of the G subfamily, three members of the K subfamily, and CqPP2C21 without the PP2Cc domain. The seven members of the G subfamily (CqPP2C2, 9, 24, 25, 67, 71, 78) have PP2C_C superfamily domain and CqPP2C10 and CqPP2C38 in the G subfamily have a PLN03145 domain belonging to the PP2C superfamily. CqPP2C8, 47, 95 in the K subfamily and CqPP2C21 have a PP2Cc superfamily domain (Serine/threonine phosphatases, family 2C, catalytic domain). Additionally, domains FHA-PP2C70-like, PKC-like superfamily, GUB_WAK_bind, MDR superfamily, ZnF-BED, CAP-ED and 2A194 superfamily also appear in quinoa PP2C protein sequences.

As is well known, the A subfamily proteins (PP2CAs) of PP2Cs are involved in controlling abscisic acid (ABA) signaling and responding to various abiotic stresses, and have a negative regulatory effect on plant growth and development. To further investigate their biological functions, a further comparison was made between the subfamily A protein of PP2Cs in quinoa and the reported PP2C proteins in *Arabidopsis*. The catalytic domain of PP2C proteins contains 11 conserved motifs, in which 5 conserved residues participate in Mg^2+^/Mn^2+^ coordination. The multiple alignment results of the 14 CqPP2C (CqPP2CA) and 9 AtPP2C (AtPP2CA) in subfamily A indicated that not all CqPP2CA members contained all the 11 conserved motifs (Figure S1). It has been found that CqPP2C94, CqPP2C108 and CqPP2C44 may lead to the elimination and loss of function of some important motifs due to the partial deletion of the N-terminus or/and C-terminus of the PP2C catalytic domain. Five sites responsible for Mg^2+^/Mn^2+^ coordination were found within all CqPP2C catalytic domains: [xxxD], [DGxG], [CGD], [DG] and [xxDN] (C-cysteine; D-aspartic acid; G-glycine; N-asparagine) (Figure S1). Subfamily A proteins of PP2C (PP2CA) had several residues involved in their phosphatase activity in the catalytic domain. Among them, the critical active-site residues [Arg138, Glu142, Asp143, Asp177, Gly178, His179, Asp347 and Asp413 in ABI1] had been conserved. Similarly, the ABA-sensing tryptophan [Trp385 (W385) in HAB1] had been conserved. Whereas, the Arg residue [Arg505 (R505) in HAB1] that mediates interaction between the ABA box and HAB1 [28] showed less conservativeness. The well-described residues responsible for ABI1-PYL1 interaction [29] were also conserved (Figure S1). These results indicated that the structures of PP2CA proteins were similar, especially within the highly conserved catalytic domain, despite greater changes in the N-terminal region (Figure S1).

### 2.4. Chromosomal Location and Duplication of CqPP2C Genes

**Figure 3:**
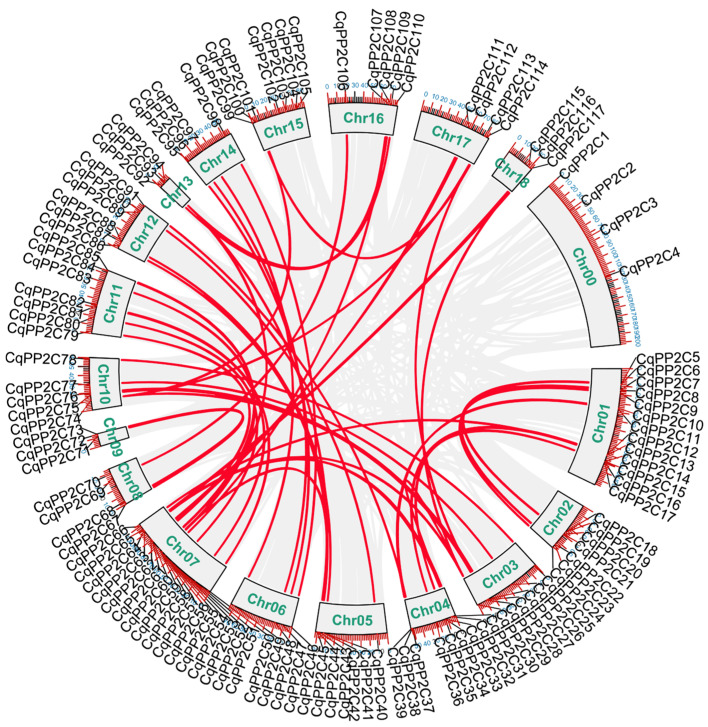
Gene location, duplication, and collinearity analysis of *CqPP2Cs*. The gray lines represent all syntenic blocks, while the red lines represent duplicate pairs of PP2C genes in the quinoa genome. The chromosome number (Chr00–Chr18) represents each chromosome.

There were a total of 113 *CqPP2Cs* located on 18 chromosomes of quinoa, and only 4 *CqPP2Cs* could not be located on any chromosome, so they were assigned to chromosome zero. According to their order on chromosomes, they were named *CqPP2C1–CqPP2C117* (Table S1 and Figure 3).

The largest number of *CqPP2C* genes were localized to chromosome 07 (17 *CqPP2Cs*), while chromosome 09, chromosome 13 and chromosome 18 had the smallest number of *CqPP2Cs* (only three *CqPP2Cs*). Gene duplication caused by polyploidization or replication-related segments and tandem duplication was the main mechanism for producing new genes, which contributes to the gene family expansion in the plant kingdom [30]. In this study, we found that there were no tandem duplication gene pairs, but 61 pairs of paralogous *CqPP2C* genes were involved in segment duplication events, indicating that segment duplication was the driving force for the expansion of the quinoa *PP2C* gene family. Among them, two *CqPP2C* gene pairs, including *CqPP2C*30/32/64/116 and *CqPP2C*29/74/101/113, had four copies. Three *CqPP2C* gene pairs, including *CqPP2C*12/13/36, *CqPP2C*31/63/117 and *CqPP2C*44/56/94, had three copies, other gene pairs contained two copies. There is only one copy of the remaining 18 *CqPP2C* genes in quinoa (Figure 3). These findings imply that gene loss could be a phenomenon within the quinoa *PP2C* gene family, leading to the elimination of certain homologous copies. Comparable patterns have been noted in the *CqWRKY* and *CqNAC* gene families in quinoa [31]. The ratio of Ka/Ks, as shown in Table S4, was consistently below 1, signifying that purifying selection predominantly drove the evolutionary dynamics of all duplicated *CqPP2C* genes.

### 2.5. Cis-Acting Elements Analysis

**Figure 4:**
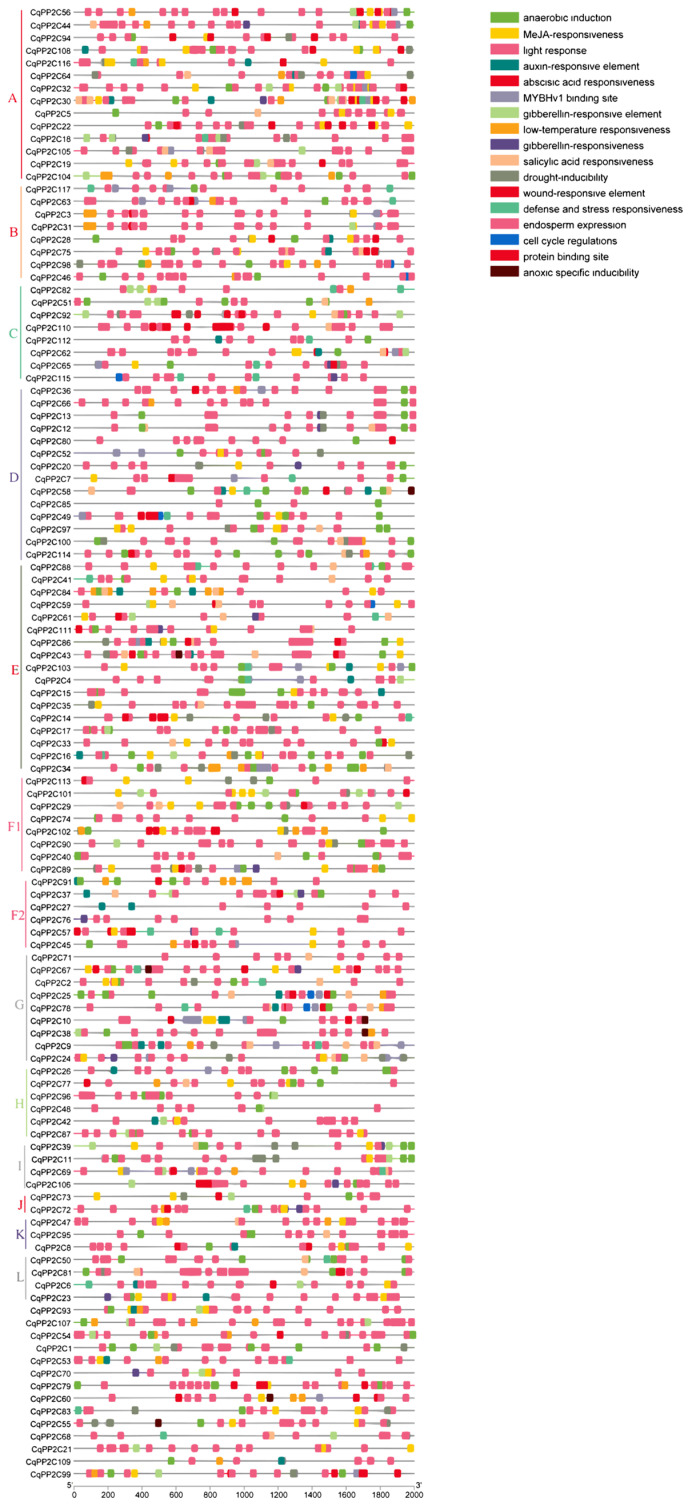
Putative cis-acting elements. The elements are displayed in differently colored boxes and their functions.

To clarify the role of the *CqPP2C* gene, a 2000 bp upstream promoter sequence of the *CqPP2C* genes was analyzed using PlantCARE. In addition to common cis-elements such as TATA boxes and CAAT boxes, other cis-elements were related to abiotic stress responses, light, hormones, plant growth and development, and other regulatory stresses (Figure 4).

According to the results, it could be seen that the number of photoresponsive elements is the largest, which was present in almost all *CqPP2C* genes. Hormone-responsive elements included abscisic acid, auxin, gibberellin, salicylic acid, and methyl jasmonate responsive elements. Abiotic stress-responsive elements included drought, anaerobic induction, low temperature, and defense and stress-responsive elements. Obviously, there were many cis-elements related to plant abiotic stress in the promoter region of the *CqPP2C* gene. Among 117 *CqPP2C* genes, 89 promoters contain ABA-responsive elements (ABRE), and *CqPP2C110* contained twelve ABA-responsive elements, with the largest number. These indicated that *CqPP2C* genes played an important role in abiotic stress responses through the ABA signaling pathway

### 2.6. Expression of CqPP2C Genes in Different Quinoa Tissues

**Figure 5:**
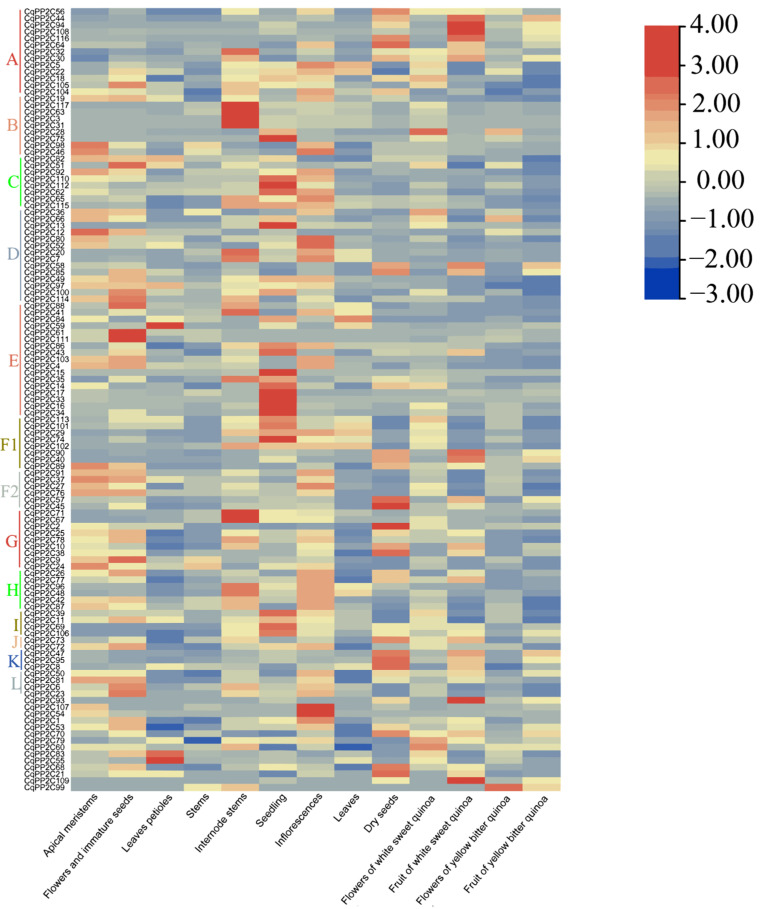
Expression patterns of *CqPP2C* genes in various quinoa tissues. This figure was drawn using TBtools.

To investigate the expression of *CqPP2C* family genes in different quinoa tissues including apical meristems, leaves petioles, flowers and immature seeds, dry seeds, stems, seedlings, internode stems, inflorescence, leaves, fruit of white sweet quinoa, flowers of white sweet quinoa, flowers of yellow bitter quinoa, and fruit of yellow bitter quinoa, a heatmap was constructed using previously published RNA-seq data. The expression levels of *CqPP2C* family genes varied in different quinoa tissues (Figure 5).

Usually, most *CqPP2C* genes are expressed in various tissues of quinoa, and several *CqPP2C* genes are prominent in certain tissues. We identified three genes with leaf petioles-specific expression (*CqPP2C55*, *CqPP2C59*, and *CqPP2C83*). *CqPP2C12* was highly expressed in apical meristems and *CqPP2C51*, *CqPP2C61*, and *CqPP2C111* accumulated in the flower and immature seeds. In addition, nine *CqPP2C* genes were abundant in internode stems and nineteen genes were prominent in the seedlings. We also found subfamily A genes of *PP2C* (*PP2CA*) were abundant in dry seeds and fruit of white sweet quinoa except *CqPP2C5*, *CqPP2C22*, and *CqPP2C105*. Through analysis, we could identify candidate *CqPP2Cs* that might play important functions in the development of different tissues.

### 2.7. Expression Patterns of CqPP2C Genes under Stress Conditions

**Figure 6:**
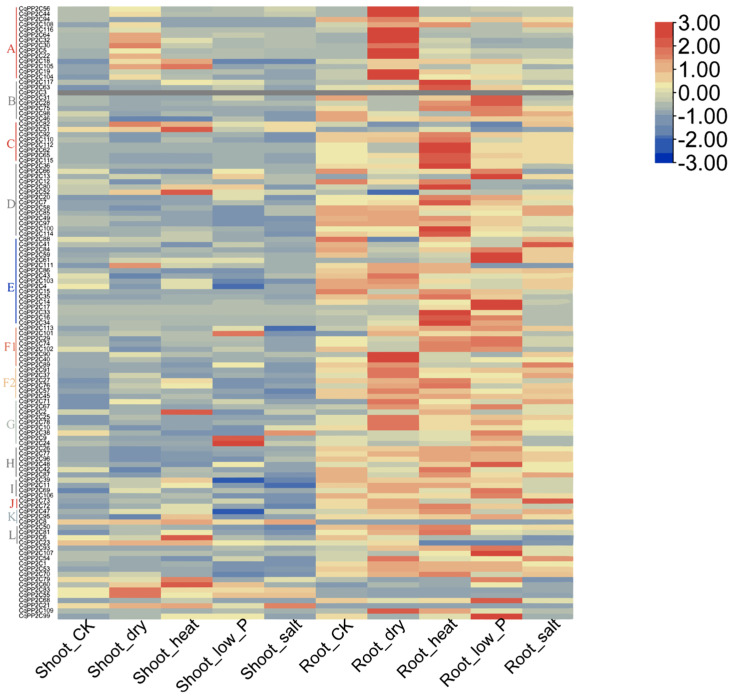
Expression patterns of *CqPP2C* genes in different treatments.

The prediction of cis-acting elements indicated that *CqPP2C* genes might be involved in the response to drought, cold, and NaCl stress. In addition, many studies have shown that *PP2C* gene expression in different plant species was regulated by abiotic stress and hormone treatment [32]. In this study, we analyzed the expression levels of *CqPP2C* genes in root and shoot under different abiotic stresses using transcriptome data (Figure 6).

The expression of *PP2C* genes in the shoot was lower than that in the root in the control. Under drought stress, compared with the control, the expression of some genes (*CqPP2C30*, *CqPP2C55*, *CqPP2C82*, and *CqPP2C83*) in shoot and genes (*CqPP2C5*, *CqPP2C19*, *CqPP2C22*, *CqPP2C32*, *CqPP2C40*, *CqPP2C43*, *CqPP2C44*, *CqPP2C56*, *CqPP2C64*, *CqPP2C90*, *CqPP2C104*, *CqPP2C109*, and *CqPP2C116*) in root significantly increased. Compared with control, some of these genes (*CqPP2C2*, *CqPP2C6*, *CqPP2C51*, *CqPP2C52*, *CqPP2C60* and *CqPP2C105*) in shoot and genes (*CqPP2C7*, *CqPP2C16*, *CqPP2C33*, *CqPP2C34*, *CqPP2C36*, *CqPP2C42*, *CqPP2C62*, *CqPP2C63*, *CqPP2C80*, *CqPP2C81*, *CqPP2C100*, *CqPP2C110*, *CqPP2C112*, *CqPP2C114*, and *CqPP2C117*) in root were significantly increased under heat stress. Some of these genes (*CqPP2C9*, *CqPP2C24*, and *CqPP2C101*) in shoot and genes (*CqPP2C13*, *CqPP2C14*, *CqPP2C17*, *CqPP2C28*, *CqPP2C31*, *CqPP2C48*, *CqPP2C59*, *CqPP2C61*, *CqPP2C68*, *CqPP2C99*, and *CqPP2C107*) in root were significantly increased under low phosphorus stress compared with control. Only *CqPP2C71*, *CqPP2C73*, *CqPP2C84*, and *CqPP2C89* in root were significantly increased under salt stress compared with control. The expression of three genes (*CqPP2C42*, *CqPP2C43*, and *CqPP2C46*) in shoot and three genes (*CqPP2C52*, *CqPP2C82*, and *CqPP2C83*) in root were significantly lower than that in control under drought stress. The expression of three genes (*CqPP2C4*, *CqPP2C39*, and *CqPP2C47*) in the shoot was significantly lower than that in control under low phosphorus. In conclusion, we found that in different treatments, most *CqPP2C* genes were highly or moderately expressed in root and shoot, indicating that most *CqPP2C* genes responded to abiotic stress.

**Figure 7:**
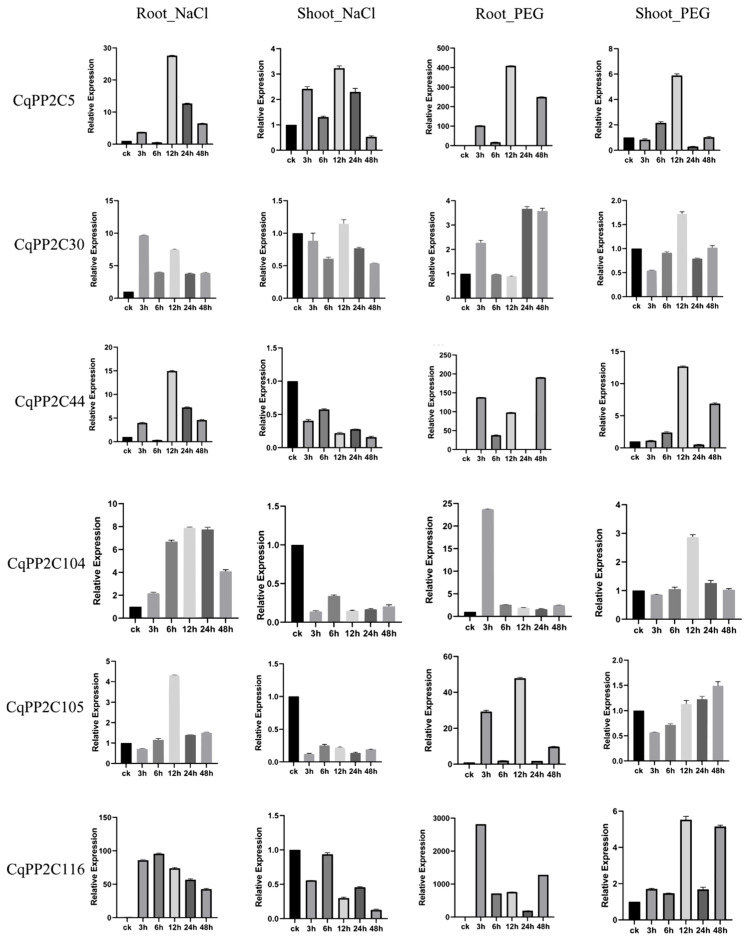
qRT-PCR was used to quantify the expression levels of 6 subfamily A *CqPP2C* genes from quinoa shoot and root under NaCl and PEG treatments. The data are an average of ±SE for three independent biological samples, and the vertical bar represents the standard deviation. Untreated shoot or root (0 h) were normalized as “1” in each graph.

It is well known that the subfamily A *PP2Cs* in rice and *A*. *thaliana* were transcriptionally regulated in abiotic stress responses dependent on ABA signaling pathways. The expression of several members of *CqPP2C* genes in subfamily A was examined by qRT-PCR under drought and salt stress in root and shoot. Six genes from subfamily A (*CqPP2C5*, *CqPP2C30, CqPP2C44*, *CqPP2C104*, *CqPP2C105*, and *CqPP2C116*) were randomly selected for this analysis (Figure 7).

The results showed that all six *CqPP2C* genes had varying degrees of response to these two stresses. Under drought treatment, six genes were up-regulated in both root and shoot, while under salt treatment, these six genes were only up-regulated in the root. In the shoot, only *CqPP2C5* was up-regulated during salt treatment, while the other five genes were down-regulated. Some results were consistent with the analysis of the transcriptome data (Figure 6). In summary, the expression patterns of subfamily A *CqPP2C* genes indicated that these genes were responsive to abiotic stress.

### 2.8. Protein Interaction between Subfamily A CqPP2Cs and CqSnRK2s

**Figure 8:**
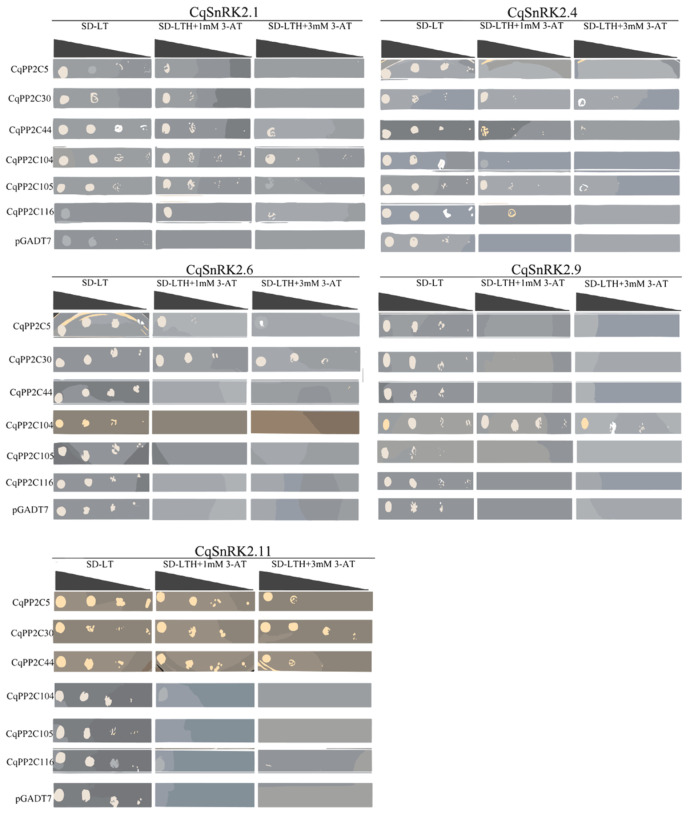
Yeast two-hybrid analysis of subfamily A CqPP2Cs and CqSnRK2s. A pGADT7 vector was used to express CqPP2Cs, and a pGBKT7 vector was used to express CqSnRK2s. Positive transformants were cultured on selective medium SD-LT (SD/-Leu/-Trp) and SD-LTH+3 AT (SD/-Trp-Leu-Ade add 3-amino-1, 2, 4-triazole) separately. The interactions of the CqSnRK2-BD constructs with pGADT7 were used as controls to test for yeast self-activation. Yeast strains were assessed at different dilution rates (1, 1/10, 1/100, and 1/1000).

The subfamily A PP2Cs (PP2CAs) has been identified to play a major negative regulatory role in the ABA signaling pathway. In this study, we studied the interactions between subfamily A CqPP2Cs and CqSnRK2s using yeast two-hybrid assay. Five CqSnRK2 members had been isolated previously in our lab, including three members of subclass II CqSnRK2s [CqSnRK2.1 (AUR62027801), CqSnRK2.4 (AUR62003254), and CqSnRK2.9 (AUR62007175)] and two members of subclass III CqSnRK2s [CqSnRK2.6 (AUR62003840) and CqSnRK2.11 (AUR62011423)]. We cloned the six subfamily A PP2C genes (*CqPP2C5*, *CqPP2C30, CqPP2C44*, *CqPP2C104*, *CqPP2C105*, and *CqPP2C116*) that had been detected for gene expression using qRT-PCR (Figure 7). As shown in Figure 8, CqPP2C104 and CqPP2C105, the homolog of AtABI1/2, could interact with CqSnRK2.1 and CqSnRK2.4, while CqPP2C104 also strongly interacted with CqSnRK2.9 but weakly interacted with CqSnRK2.11. CqPP2C5, CqPP2C30, and CqPP2C44 strongly interacted with CqSnRK2.11 and weakly interacted with CqSnRK2.1 and CqSnRK2.4.

In addition, the homolog CqPP2C30 of AtAHG3 strongly interacted with CqSnRK2.6, while the homolog CqPP2C116 of AtHAI1/2/3 only weakly interacted with CqSnRK2.1 and CqSnRK2.4. The results showed that the six subfamily A CqPP2Cs exhibited complex interactions with the five CqSnRK2s.

## 3. Discussion

Plant PP2Cs play a crucial role in governing various essential biological processes associated with development and stress response [33,34]. In the present study, we conducted a thorough analysis of the *CqPP2C* genes in *C. quinoa*, identifying a total of 117 *CqPP2C* genes. Compared with *Arabidopsis thaliana* (80), rice (78) [35], *Brachypodium distachyon* (86) [4], *Medicago truncatula* (94) [36], cucumber (56) [37], tomato (56) [38], and maize (97) [39], the amount of PP2C in quinoa was much more. Although the genome sizes of higher plants such as rice and *Arabidopsis* are comparable to those of lower plants such as green algae (*Chlamydomonas reinhardtii*), lycophyte (*Selaginella moellendorffii*) and moss (*Physcomitrella patens*), there are only 10 *PP2C* genes in green algae, and 50 *PP2C* genes in lycophyte and moss, while 78 in rice and 80 in *Arabidopsis* [40]. This indicated that there were differences in the expansion of the PP2C genes among different species, which may be related to the evolution of plants from unicellular organisms to multicellular organisms.

The *PP2C* gene of quinoa was organized into 13 subfamilies by branches of the phylogenetic tree (Figure 1), consistent with the *PP2C* groups in *A. thalinan*, *Oryza sativa* [35], *B. distachyon* [4], and cucumber [37]. In phylogenetic analysis, different PP2C groups of quinoa and *Arabidopsis* were arranged together to form a common branch, indicating that PP2C had sequence conservation and similar evolutionary lineages. Phylogenetic analysis can identify homologous genes from different species to predict gene function. In the A subfamily, the CqPP2C18, CqPP2C19, CqPP2C104 and CqPP2C105 protein was homologous with AtABI1 (AT4G26080) and AtABI2 (AT5G57050), indicating that these four CqPP2C proteins might be involved abiotic stress in plants and were believed to have a negative regulatory effect on ABA signaling [41,42]. Similarly, the AtPP2CF1 (AT3G05640) protein in the E subfamily can activate cell proliferation and expansion, as well as accelerate inflorescence growth, and its homologous CqPP2C41 and CqPP2C88 may have the same function [43].

The exon/intron structure of genes and protein-conserved motifs are important markers of the evolutionary relationship of family genes. Accordingly, we analyzed gene structure and protein-conserved motifs of CqPP2Cs (Figure 2). The findings revealed a consistent exon/intron structure among *CqPP2Cs* belonging to the same subfamily, with some exceptions, which might be due to different reasons. Previous studies on *Brachypodium distachyon*, *Fragaria vesca*, and *Fragaria ananassa* had shown that there were many *PP2C* genes with intron deletion [4,44], and similar results had been found in quinoa research. Twenty conserved motifs were identified. As shown in Figure 2B, CqPP2Cs in the same subfamily exhibited similar motif distribution. When analyzing conservative domains, in addition to the main PP2C phosphatase domain, we also found 14 other domains. KAPP (kinase-associated protein phosphatase) is an *Arabidopsis* PP2C that contains FHA (forkhead associated domain) at the N-terminus of its kinase interaction region, which is crucial for connecting to phosphorylated target proteins and thus facilitates signal transduction [45]. Therefore, studying the important functions of CqPP2Cs carrying these special structural domains would be of interest.

The *CqPP2C* gene exhibits a tissue-specific spatial expression pattern. The abundant presence of the PP2C subfamily A gene in the dry seeds and fruit of white sweet quinoa indicated that these genes are involved in ABA-mediated seed development, dormancy, and germination [46]. Cis-acting elements are important regulators of resistance to various stresses and hormone responses in plant development. We found that ABREs and DREs (Drought response elements) elements were abundant in most *CqPP2C* gene promoter regions, indicating that *CqPP2C* genes may play an indispensable role in the ABA signaling pathway, acting on drought resistance or salt stress resistance. Further expression analysis showed that most *CqPP2Cs* responded to drought, heat, salt stress, and Pi starvation. As is well known, the subfamily A PP2Cs plays an important role in the ABA signaling pathway and plant response to abiotic stress [47,48]. We identified fourteen *PP2C* genes belonging to the A subfamily in quinoa through sequence alignment and evolutionary analysis. We found that most members of the A subfamily in quinoa were significantly up-regulated or down-regulated under drought and/or salt stress, which was consistent with reports from other plants. AtABI1 and AtABI2 have been identified as important components in the ABA signaling pathway [40,47,48]. *CqPP2C64* and *CqPP2C116*, which are homologs of *AtHAI* PP2Cs, were significantly up-regulated under drought stress. *AtHAI* PP2Cs had unique drought resistance functions in *Arabidopsis*. The *HAI PP2C* mutant reduced the expression of several defense-related genes under low water potential but increased the expression of abiotic stress-related genes encoding late embryogenesis abundant proteins and dehydratin, as well as increased the accumulation of proline and osmoregulatory solutes [48]. Likewise, the expression of *BdPP2Cs* in *B. distachyon* and *MtPP2Cs* in *M. truncatula* from subfamily A were induced by cold, heat, drought, salt, or H_2_O_2_ treatment [4,36]. In *P. euphratica*, ABA has a moderate inducing effect on *PeHAB1*, while drought stress has a significant inducing effect on *PeHAB1* [49]. Otherwise, the expression of *TaPP2C59* in wheat and most *FvPP2Cs* in *F. vesca* were significantly down-regulated under drought and high salt stress, suggesting that these genes play a negative regulatory role [44]. Most members of subfamily A AtPP2Cs have been identified as negative regulators of ABA signaling. ABA treatment and abiotic stress can highly induce the expression of these genes [35,50]. The induction of *PP2C* gene expression by ABA might be the ABA desensitization mechanism that regulates ABA signaling and maintains plant homeostasis.

Similar to the research results of other plants, our study in quinoa also indicated that some *CqPP2Cs* from subfamilies other than subfamily A were induced by abiotic stresses. In the B subfamily, the expression of *CqPP2C63* and *CqPP2C117* was highly induced under heat stress, and the expression of *CqPP2C28* and *CqPPS2C31* was significantly altered after Pi starvation treatment in root. The expression of *CqPP2C52* in the shoot and the expression of *CqPP2C7*, *CqPP2C36*, and *CqPPS2C31* in the root were highly induced under heat stress. A study has shown that almost all members of subfamily D in *Arabidopsis* and soybean contain heat stress response elements (HSE) in their promoters, and subfamily D genes in wheat respond to heat treatment [19,51]. These results indicate that CqPP2Cs can play important functions under different stresses, and their detailed roles still need further exploration.

In *Arabidopsis*, members of subfamily A PP2Cs can interact with both ABA receptor PYLs and subclass III SnRK2s, mediating the ABA signaling pathway to regulate seed germination and response to abiotic stress [52,53]. Subclass III AtSnRK2 (AtSnRK2.2, AtSnRK2.3, AtSnRK2.6) proteins always interact with subfamily A PP2Cs and are inactivated by direct dephosphorylation of subfamily A PP2Cs [52,54]. In our study, the results of the yeast two-hybrid assay showed that all six CqPP2Cs interacted with one or two members of CqSnRK2s. Subfamily A CqPP2Cs interacted not only with subclass III CqSnRK2s but also with subclass II CqSnRK2s. This result was consistent with studies in rice, *B. distachyon*, and wheat. In rice, OsSAPK2 was classified as Class 2b (subclass II) and could interact with OsPP2C30 [55]. In *B. distachyon*, group A BdPP2C could interact with subclass II BdSnRK2.1 [56], and group A TaPP2C interacted with subclass II TaSnRK2s in wheat [51]. This suggested that subfamily A CqPP2Cs were essential for ABA signal transduction in quinoa, where subfamily A CqPP2Cs bound to CqSnRK2s in different combinations and intensities to respond to various biological processes and stresses.

## 4. Materials and Methods

### 4.1. Identification of the PP2C Gene Family in Quinoa

This experiment used BLASTP and the Hidden Markov Model (HMM) [57] to identify the *PP2C* genes in quinoa. The sequences of 80 AtPP2C proteins were downloaded from the website (https://www.arabidopsis.org/, accessed on 7 June 2022). The quinoa genome data (http://www.cbrc.kaust.edu.sa/chenopodiumdb/, accessed on 7 June 2022) were used for BLASTP and the retrieval threshold was set as E-value < E^−10^. To further screen candidate genes, we compared the protein sequences of *Arabidopsis* PP2C using TBtools. Then, the NCBI-CDD databases (https://www.ncbi.nlm.nih.gov/cdd/, accessed on 7 June 2022) and Pfam (http://pfam.xfam.org/search#tabview=tab1, accessed on 7 June 2022) [58] were used for domain identification of candidate gene. We manually deleted candidate genes without specific domains of the PP2C (registration numbers PF00481, PF07830, and PF13672) [51]. The physicochemical properties were analyzed based on ExPASy (https://web.expasy.org/protparam/, accessed on 7 June 2022) [59]. Online software WoLF PSORT (http://www.genscript.com/wolf-psort.html, accessed on 7 June 2022) predicted the subcellular localization of CqPP2Cs [60].

### 4.2. Evolutionary Relationship of the PP2C Gene Family

Validated quinoa PP2C protein sequences and *Arabidopsis* PP2C protein sequences (AtPP2Cs) were used to establish an evolutionary relationship. This analysis included a total of 197 amino acid sequences. Using MEGA 7 to align multiple protein sequences, the final comparison results were constructed through neighbor-joining (NJ), and the bootstrap value was set to 1000 [35,37]. The constructed tree was beautified using iTOL (https://itol.embl.de/, accessed on 7 June 2022).

### 4.3. Gene Structure and Protein Conserved Motif Analysis

Conserved motifs of CqPP2Cs were determined using MEME (http://meme-suite.org/tools/meme, accessed on 7 June 2022) for all CqPP2C sequences. The number of motifs was set to 20, and other parameters were defaults [61]. We extracted gene structures from genome annotation gff3 files and used TBtools to display the results. DNAMAN v.8.0 software was used for CqPP2Cs amino acid multiplex sequence alignment.

### 4.4. Chromosomal Location and Gene Duplication Analysis

TBtools-II (Toolbox for Biologists) v2.003 software was utilized to perform synteny analysis within the quinoa genome by employing all-vs-all BLASTP alignments. The plugin MCScanX method was employed for this analysis [62]. Duplication events and synteny analysis were visualized using TBtools software [63]. Additionally, TBtools was utilized to calculate the synonymous (Ks) and nonsynonymous (Ka) substitution rates of homologous genes in quinoa [63].

### 4.5. Analysis of Cis-Acting Elements in the Promoter Regions

The promoter sequence (2000 bp upstream of the putative genes ATG) was extracted using TBtools. Then submitted the promoter sequence to PlantCARE (http://bioinformatics.psb.ugent.be/webtools/plantcare/html/, accessed on 7 June 2022) for cis-element prediction [64].

### 4.6. Analysis of CqPP2C Gene Expression Patterns

The quinoa RNA-seq data from different tissues (No: PRJNA394651) and different treatments (No: PRJNA306026) were downloaded from the Bioproject database (http://www.ncbi.nlm.nih.gov/sra, accessed on 7 June 2022). RNA-seq data in TPM (transcripts per million reads) 491 is normalized and a log_2_ transformation is performed. We used TBtools software to visualize the heatmap of *PP2C* gene expression.

### 4.7. Quinoa Treatment and RNA Extraction

The quinoa material was “YT077”. Quinoa was cultivated in a greenhouse (with an average temperature of 22 °C, relative humidity of 70–75%, and 16 h/8 h of light/dark). To determine the expression of quinoa *PP2C* under salt and drought treatment. One-month-old quinoa seedlings with consistent growth were selected for salt stress (300 mM NaCl) and drought stress (20% PEG6000). The leaves and roots of seedlings were taken at 0 h, 3 h, 6 h, 12 h, 24 h and 48 h after treatment. The collected samples were frozen quickly with liquid nitrogen and placed at −80 °C until further usage. Three biological replicates for each treatment. TransZol Up Plus RNA Kit (Transgen, Beijing, China) was used to extract total RNA from collected samples.

### 4.8. qRT-PCR Analysis

The primers were designed using Primer3 (http://bioinfo.ut.ee/primer3-0.4.0/, accessed on 9 June 2022) (Table S5), synthesized by Qingdao BGI. The above preserved RNA was reverse-transcribed into single-stranded cDNA using the TransScript^®^ One-Step gDNA Removal and cDNA Synthesis SuperMix (Transgen, Beijing, China). The cDNA product obtained by reverse transcription was used as a template, and the SYBR Green I dye method was used for real-time PCR, and the quinoa *Tubulin* gene was used as the internal reference gene [65,66]. The reaction volume was 20 μL, containing 2 μL of cDNA solution, 2 μM forward and reverse primers, 10 μL of SYBR, and 6 μL of deionized water. The amplification program conditions were as follows: pre-denaturation at 95 °C for 30 s; Denaturation at 95 °C for 10 s; Annealing at 57 °C for 15 s; Extension at 72 °C for 30 s and set the number of cycles to 40 times. The relative expression of genes was calculated by using the 2^−ΔΔCT^ method [67].

### 4.9. Yeast Two-Hybrid Assays

The primers (Table S6) for the cloning of *CqPP2Cs* and *CqSnRK2s* were designed using the Primer3. Six *CqPP2Cs* in group A and five *CqSnRK2s* were amplified from the quinoa cDNA. The *CqPP2C* and *CqSnRK2* genes were cloned into pGADT7 and pGBKT7 vectors, respectively. Yeast two-hybrid analysis was conducted with the yeast strain AH109, following the manufacturer’s protocol from Clontech, USA, and the experiment was repeated at least three times. Positive transformants screened from the SD medium lacking leucine and tryptophan (SD/-Leu/-Trp) are then transferred to the SD medium lacking leucine, tryptophan, and histidine (SD/-Leu/-Trp/-His) for further screening. The 3-amino-1, 2, 4-triazole (3-AT) was used to eliminate the background yeast growth on SD medium lacking leucine, tryptophan, and histidine (SD/-Leu/-Trp/-His).

## 5. Conclusions

In the present work, a total of putative 117 *CqPP2C* genes were identified and divided into 13 subfamilies. The chromosome localization, physical and chemical feature predictions, phylogenetic analysis, gene structure, conserved motif and domain analysis were thoroughly investigated. The results of collinearity and selection pressure analysis indicated that *CqPP2C* genes underwent amplification of segmental duplication and purification selection during evolution. In addition, the expression of *CqPP2Cs* in various tissues under different abiotic treatments was analyzed using RNA-seq data. *CqPP2C* genes were involved in regulating the development and stress responses of quinoa. qRT-PCR results showed that six *CqPP2C* genes in subfamily A were up-regulated or down-regulated under salt and drought treatments. Additionally, Yeast two-hybrid assays showed that subfamily A CqPP2Cs interacted with CqSnRK2s in different combinations and intensities to respond to various biological processes and stresses. Overall, our results provide new insights and a basis for further understanding the roles of the CqPP2C family in the regulation of abiotic stress response.

## Data Availability

The data presented in this study are available in the article and Supplementary Materials.

## Supplementary Materials

The following supporting information can be downloaded at: https://www.mdpi.com/article/10.3390/genes15010041/s1, Figure S1: Amino acid sequence and secondary structure alignment of subfamily A PP2C proteins in quinoa and *Arabidopsis* with 11 conserved motifs (A–K). The black arrowheads below the sequence indicate the active-site residues. The red circles below the sequence indicate the residues involved in the interaction with PYL1. The blue arrowhead indicates the conserved Gly residue. The green arrowhead indicates the conserved ABA-sensing tryptophan. The yellow arrowhead indicates the conserved Arg residue [Arg505 (R505) in AtHAB1] that mediates interactions between HAB1 and the ABA box; Table S1: The list of 117 *CqPP2C* genes and their basic characterizations; Table S2: The distribution of *PP2C* genes in *Arabidopsis* and quinoa; Table S3: Conserved motifs in the amino acid sequences of CqPP2C proteins; Table S4: Ka/Ks of syntenic gene pairs in quinoa genome; Table S5: Primers used for qRT-PCR in this study; Table S6: Primers for plasmid construction.
