# Supplementary material for: Genome-Wide Identification, Expression and Interaction Analyses of PP2C Family Genes in Chenopodium quinoa
Source: Genes (Basel). 2023 Dec 27;15(1):41. doi: 10.3390/genes15010041 (PMC10815568; doi:10.3390/genes15010041)
Supplement: Supplementary file 1 [file genes-15-00041-s001.zip › Captions for SUPPLEMENTARY MATERIAL.pdf]

Figure S1 Amino acid sequence and secondary structure alignment of subfamily A PP2C proteins in quinoa and *Arabidopsis* with 11 conserved motifs(A-K). The black arrowheads below the sequence indicate the active-site residues. The red circles below the sequence indicate the residues involved in the interaction with PYL1. The blue arrowhead indicates the conserved Gly residue. The green arrowhead indicates the conserved ABA-sensing tryptophan. The yellow arrowhead indicates the conserved Arg residue [Arg505 (R505) in AtHAB1] that mediates interactions between HAB1 and the ABA box.

Table S1 The list of 117 *CqPP2C* genes and their basic characterizations.

Table S2 The distribution of *PP2C* genes in *Arabidopsis* and quinoa.

Table S3 Conserved motifs in the amino acid sequences of CqPP2C proteins.

Table S4 Ka/Ks of syntenic gene pairs in quinoa genome.

Table S5 Primers used for qRT-PCR in this study.

Table S6 Primers for plasmid construction
