# Supplementary material for: Genome-Wide Identification, Expression and Interaction Analyses of PP2C Family Genes in Chenopodium quinoa
Source: Genes (Basel). 2023 Dec 27;15(1):41. doi: 10.3390/genes15010041 (PMC10815568; doi:10.3390/genes15010041)
Supplement: Supplementary file 1 [file Figure S1. Amino acid sequence and secondary structure alignment of subfamily A PP2C proteins in quinoa and Arabidopsis.pdf › Figure S1. Amino acid sequence and secondary structure alignment of subfamily A PP2C proteins in quinoa and Arabidopsis.pdf]

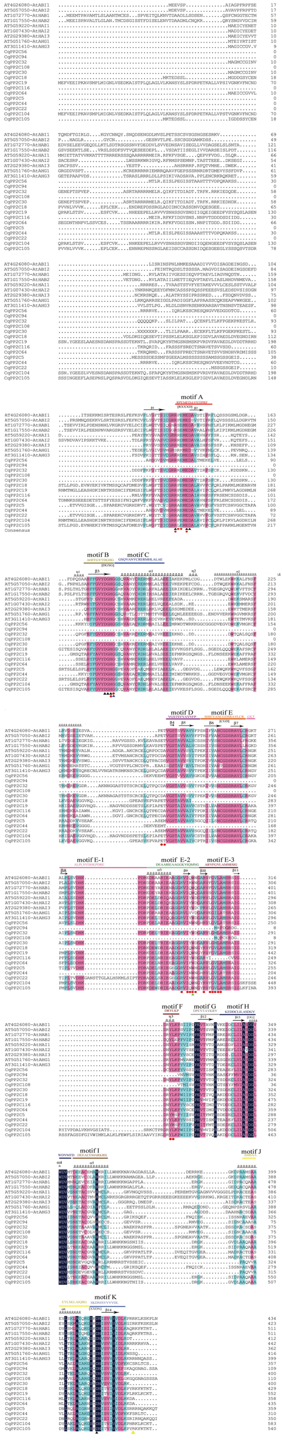
motif A
motif B
motif C
motif D
motif E
motif E-1
motif E-2
motif E-3
motif F
motif G
motif H
motif I
motif J
motif K
Consensus
